# **Genetics and**”**democracy**”

**DOI:** 10.1186/s13052-022-01391-7

**Published:** 2022-12-26

**Authors:** Federico Marchetti, Giovanni Corsello

**Affiliations:** 1 Department of Pediatrics, Santa Maria Delle Croci Hospital, Viale Randi 5, 48121 Ravenna, Italy; 2 Department of Sciences for Health Promotion and Mother and Child Care ”G. D’Alessandro”, University of Palermo, Palermo, Italy

**Keywords:** Genetics, Children, Screening, Ethics, Democracy

## Abstract

**Background:**

The spread of knowledge on the important implications of a diagnosis of genetic disease does not correspond to a sharing of the knowledge and equal rights of children.

**Main body:**

It is estimated that about 5% of newborns may have a rare disease that in some cases, if diagnosed early, could have specific treatments that may be able to modify the natural history of the disease. However, in most countries the diagnosis during the first hours of life is limited to a few diseases, due to the high costs and time required for genetic investigations with classical methods. Recently, experimental projects to subject all newborns to a complete DNA analysis, with Next Generation Sequencing techniques, to detect any genetic pathologies as early as possible, have been reported in some countries. The late diagnosis of some genetic diseases that have treatment plans, such as spinal muscular atrophy, can be a serious damage, for anyone who has seen and accompanied the life of a child with this disease and his/her family, before and after, the recent availability of therapies which, if started very early, can lead to an almost normal life. Rapid sequencing and genetic diagnosis are a crucial part of directing inpatient management and this resource should be accessible not only to academic medical centers but also in community settings.

**Conclusions:**

It is time for a profound reflection that places in Italy, as in other countries, the use of genetic tests in neonatal and pediatric age based on principles of evidence, ethics, and democracy and on clear national guidelines, which also consider organizational aspects.

## Background

The full knowledge of human genome at the beginning of present century has increased in people the hope of applying genetic testing to diagnose and predict all genetic diseases [1]. It is now well known that human beings are the product of a mixture of genes, environment and epigenetic markers imprinted in the early phases of development. In this perspective there is no coincidence between genomic asset and clinical consequences, particularly for multifactorial as well as polygenic diseases. The relationship between genetics and ethics has been much discussed during the last decade, while the relationship between genetics and the political arena—with terms such as rights, distribution, expertise, participation, and democracy—has been less considered [2].

Nevertheless, at the end of 2021, concrete discussions began in England and the United States of subjecting all newborns to a complete DNA analysis immediately after delivery to discover any genetic pathologies as early as possible [3–5]. The resulting debate continues to be very intense from an ethical and scientific point of view.

### Main findings

Proponents of complete DNA mapping point out that a not small percentage of children (from 5 to 7%) are born with a rare disease, sometimes treatable, or in any case well controllable if intervened early [5]. However, in most countries the diagnosis during the first hours of life, or even during pregnancy, is limited to a few diseases, due to the high costs and time required for genetic investigations with classical methods.

Proponents of genomic screening argue that with techniques known as Next Generation Sequencing (NGS), entire genomes can be “deciphered” in hours, at affordable costs. Having all the genetic information at birth—say the proponents of mass screening—could bring great benefits and translate into considerable savings, ensured by the fact of not having to treat disabilities and diseases over the course of life. Based on this principle, Genomics England (a company created by the UK Department of Health and Welfare) has launched a pilot study on 200,000 children, where anomalies associated with over 600 rare diseases will initially be checked, all curable, at least partially [5]. The DNA will be stored to allow further investigations in the future, such as those on the predisposition to cancer, or on the effectiveness of specific drugs.

In the United States, plans are being made to launch a similar program to prevent or treat—when possible—the most dangerous rare diseases, but the times will certainly be longer [5].

The two projects have significant organizational differences, which could have important consequences, as reported in a dedicated issue on this new frontier in the journal *Science* [5]. In Great Britain, in fact, everything is in the hands of Genomics England, an agency that has been in operation for some time, with well-standardized criteria. Each DNA sample will be collected, treated, and stored in the same way by a network of laboratories connected to each other, following specific guidelines, and responding to strict limits relating to the privacy and ethics of what is offered to families. In the USA, each state will provide autonomously, also relying on private structures, with the risk of a multiplication of different DNA deciphering systems. In this type of test management, ethical guidelines may also become highly variable. The debate also raises many doubts about the effectiveness of the tests themselves [6], which would give inaccurate responses in more than one case out of ten, with possible risks of overdiagnosis and what this can entail for families is easily imaginable [7]. Some studies that asked parents' opinion on the genetic screening of their newborns did not have a univocal answer in a favorable sense, indeed many parents did not declare themselves willing to accept these forms of preventive genetic screening [5].

In 2018, the U.S. National Institutes of Health issued a document reiterating that there is still not sufficient justification for carrying out wide-ranging genetic tests on all children, because the effects of many mutations are not known, and why many genetic diseases lack therapy [5]. On the contrary, some patient and medical associations argue that the already validated tests should be implemented much better, and their dissemination should be encouraged, making procedures faster. Supporters of the complete genetic mapping of newborns reply by reporting some evidence: according to what emerged from 23 studies, for example, the expanded genetic investigations led to an early diagnosis of rare diseases in 36% of 1839 children (mainly infants), with the possibility of activating a therapy in 29% of cases, sometimes lifesaving. "Genome sequencing—reports the journal Science—is becoming a new form of care for critically ill infants"[5].

The precise information aspect of what we are looking for is decisive when we need to talk to a parent about genetics and what we ask ourselves is whether we are ready for such relevant information (a consent) if we are talking about NGS which cannot be individual to moment of conception, but that must start well earlier, at the population level. The debate, on the other hand, seems very confined to laboratories and research groups, praiseworthy in scientific progress (trouble if not) but which do not have the aptitude to tackle the very serious problem of knowledge that becomes participatory and democratic, even in the method, on the impact of a diagnosis, on the risks of false positives (or negatives) and on the true possibility of cure, also knowing that for some therapies (sometimes still experimental and as such unauthorized) the knowledge on the risks and benefits are not fully known. The impact of NGS on connecting patients with rare diagnoses through social media or internet-based resources can be explore more thoroughly [5]. Rapid sequencing and genetic diagnosis are a crucial part of directing inpatient management, and this resource should be accessible not only to academic medical centers but also in community settings [8].

The late diagnosis of some genetic diseases that have treatment plans, such as spinal muscular atrophy (SMA), can be a serious damage, for anyone who has seen and accompanied the life of a child with this disease and his / her family, before and after, the recent availability of therapies which, if started very early, even in the forms of SMA type 1, can lead to an almost normal life [9]. Without these innovative therapies we are facing a certain and early death or a serious disability, even for the less severe forms of SMA. But now in fact, in Italy, early neonatal screening for SMA is done only in the Lazio and Tuscany region [10] and it has been debating for some time about when it will be extended to the whole country. And still, in case of suspicion of SMA (which must be as early as possible in front of a hypotonic newborn/infant), what we see is that depending on the laboratories scattered in Italy, the answers could be very different: from a few days to several days. And the times could be even longer if, faced with a reasonable suspicion, a family or hospital pediatrician were to turn to a genetic examination for the execution of the test which is often not direct but must go through the advice of other specialists. The scenario, in the common welfare realities, is a problem that is faced with patchy responses according to the contexts of care.

Another illustrative example on the doubts and potential of a genetic diagnosis concerns the autism spectrum disorder (ASD). Many experts tell us that all children with a certain diagnosis of ASD (2% of newborns!) should undergo a genetic screening with comparative genomic hybridization (CGH)-array (as well as the karyotype for fragile X syndrome) [11]. Others are advocates of screening only in the presence of an indicative phenotype for genetic disease. But the question we often ask ourselves is what the state of things in Italy is, namely whether all children diagnosed with ASD are subjected to genetic screening (we would say regardless of the geneticist's evaluation once the diagnosis has been made or strongly suspected). Experience in the field, on the other hand, speaks of very long waiting times, not so much and not only for genetic evaluations and investigations (which are of relatively little interest to parents at that moment in the clinical diagnosis) but also and above all in therapeutic treatment.

## Conclusions

It is time for a profound reflection that places the use of genetic tests in neonatal and pediatric age based on principles of evidence, ethics, and democracy and on clear national guidelines, which also consider organizational aspects. What we would like as pediatricians attentive to the progress of genetics, is that a serious debate opens in Italy with very pragmatic implications (waiting for difficult and perhaps distant decisions on a genomic population screening with NGS) and that it decides on:

a) The opportunity for a very defined expanded neonatal screening that is the same for all Italian newborns, and that considers those diseases that, thanks to highly sensitive and specific assessment methods, have an immediate plan of care. He will not be able to understand all of what is known, but the example of SMA is emblematic, for genetics that is indeed democratic.

b) A guide to the services of public genetics laboratories, again on a national basis, which can guarantee, beyond newborn screening, rapid and competent responses to specific pathologies they have (according to the opinion of expert clinicians), a high suspicion for genetic diseases with important therapeutic implications (see genetic epilepsies, autoinflammatory diseases and several others).

c) Organizational practical times, these are on a regional basis or in wider fields (but known and well organized), to provide services that are, when necessary, of rapid advice, in close interface with the pediatricians who are treating that single case and family.

## Data Availability

Not applicable.

## Abbreviations

ASD: Autism spectrum disorder
NGS: Next Generation Sequencing
SMA: Spinal muscular atrophy
